# Efficacy of tailored second-line therapy of *Helicobacter pylori* eradication in patients with clarithromycin-based treatment failure: a multicenter prospective study

**DOI:** 10.1186/s13099-020-00378-1

**Published:** 2020-08-29

**Authors:** Siya Kong, Keting Huang, Jun Wang, Xiaoyong Wang, Ningmin Yang, Yu Dong, Ya Zhuang, Yini Dang, Guoxin Zhang, Feng Ye

**Affiliations:** 1 Department of Gastroenterology, First Affiliated Hospital of Nanjing Medical University, No. 300 Guangzhou Road, 210029 Nanjing, China; 2 First Clinical Medical College of Nanjing Medical University, Nanjing, China; 3 Department of Gastroenterology and Hepatology, Jinhu County People’s Hospital, Huaian, China; 4 Department of Gastroenterology, Changzhou Second People’s Hospital Affiliated to Nanjing Medical University, Changzhou, China; 5 Jiangsu Zhiyuan Inspection Medical Institute, Nanjing, China

**Keywords:** Tailored therapy, Clarithromycin resistance, Second-line therapy, Levofloxacin, *Helicobacter pylori*

## Abstract

**Background:**

After the failure of clarithromycin- and bismuth-based quadruple therapy (CBQT), levofloxacin- and bismuth-based quadruple therapy (LBQT) is recommended for *Helicobacter pylori* eradication. We compared the efficacies of second-line tailored bismuth-based quadruple therapy (TBQT) and empirical LBQT.

**Methods:**

Patients with CBQT failure were randomly assigned to receive TBQT or LBQT for 14 days. All patients underwent endoscopy for culture-based antibiotic susceptibility testing. Patients in the TBQT group exhibiting levofloxacin susceptibility were randomized to receive amoxicillin, levofloxacin, esomeprazole, and colloidal bismuth pectin (ALEB) or amoxicillin, furazolidone, esomeprazole, and colloidal bismuth pectin (AFEB) for 14 days; patients with levofloxacin resistance received AFEB.

**Results:**

From May 2016 to June 2019, 364 subjects were enrolled. Eradication rates were significantly higher in the TBQT group (n = 182) than in the LBQT group (n = 182) according to both intention-to-treat (ITT) analysis (89.6% vs. 64.8%, P < 0.001) and per protocol (PP) analysis (91.1% vs. 67.8%, P < 0.001). Among patients in the TBQT group with levofloxacin susceptibility, eradication rates were similar in the ALEB (n = 51) and AFEB (n = 50) subgroups according to both the ITT (86.3% vs. 90.0%, P = 0.56) and PP (88.0% vs. 90.0%, P = 0.75) analyses. Isolated clarithromycin and levofloxacin resistance rates were 57.7% and 44.5%, respectively. The total clarithromycin and levofloxacin resistance rate in strains with dual or triple resistance was 35.7%.

**Conclusions:**

TBQT was more effective than LBQT as a second-line strategy after CBQT failure. In the absence of antibiotic susceptibility testing, AFEB therapy might be used as a rescue therapy to eradicate *H. pylori* and avoid levofloxacin resistance.

Trial registration: Chinese Clinical Trial Registry (www.chictr.org.cn): ChiCTR1900027743.

## Background

*Helicobacter pylori* (*H. pylori*) infection, a bacterial infection of the stomach, affects approximately 50% of the global population [1]. *H. pylori* infection is associated with risks of peptic ulcer, chronic gastritis, intestinal metaplasia, and gastric cancer [2]. Therefore, consensus reports have proposed that *H. pylori* eradication treatment is necessary to prevent these diseases [2, 3], particularly among patients who have a family history of gastric cancer in first-degree relatives [4]. Unfortunately, the eradication rates of standard therapy have decreased to alarmingly low levels due to increasing levels of antibiotic resistance; eradication is particularly difficult when the clarithromycin resistance rate is greater than 15% in the local region [5, 6].

In recent decades, the resistance rates to clarithromycin, levofloxacin, and metronidazole have reached 22%, 19% and 78%, respectively, in China [7]. The prevalence of resistance to clarithromycin (22.9–37%), levofloxacin (5.7–34.6%), and metronidazole (14.4–93.2%) has increased significantly over time and varies substantially among countries in the Asia–Pacific region [8, 9]. The success rate of standard *H. pylori* eradication treatment has decreased to 60% due to increasing levels of unrecognized antibiotic resistance, high intragastric bacterial loads before treatment, poor compliance, and the rapid metabolism of proton pump inhibitors (PPIs) [5, 10]. The use of antibiotic susceptibility-guided therapy has therefore been proposed as a countermeasure for resistance-associated treatment failure and the emergence of antibiotic resistance [11]. In regions with a high clarithromycin resistance rate (26.12%), the administration of tailored colloidal bismuth pectin-containing quadruple therapy to patients for 14 days has achieved eradication rates of 85.99% and 91.22% according to intention-to-treat (ITT) and per protocol (PP) analyses, respectively [12]. The eradication rates of susceptibility-guided therapies have been reported to exceed 90%, according to PP analyses, in regions with high clarithromycin resistance rates (> 15%), even when these therapies were used as rescue treatments [2, 11–14].

After the failure of clarithromycin-based triple therapy, colloidal bismuth pectin-containing quadruple therapy or fluoroquinolone-containing triple or quadruple therapy is recommended as a second-line treatment by the Maastricht V/Florence Consensus Report [15]. However, patients with treatment failure are more likely to be infected with metronidazole-, levofloxacin-, and/or clarithromycin-resistant bacteria [16, 17]; thus, the efficacy of levofloxacin has decreased in recent years. Hence, an improved rescue therapy regimen for *H. pylori* infection following the failure of first-line clarithromycin-containing eradication treatment is needed. Based on data from pilot studies, the administration of tailored treatment as a second-line rescue treatment for *H. pylori* infection achieved a very high eradication rate (95%) [11]. However, the benefits of tailored second-line treatment remain unclear [15, 18]. Currently, researchers have not yet determined whether the tailored quadruple regimen is superior to standard levofloxacin-containing quadruple therapy, and its efficacy as a rescue treatment for patients with failure of clarithromycin-based quadruple therapy remains to be determined. Therefore, in this prospective, randomized clinical trial, we evaluated the possibility of using levofloxacin- or furazolidone-based quadruple therapy as a universal rescue treatment. In addition, we investigated the efficacy of tailored bismuth-based quadruple therapy (TBQT; containing levofloxacin or furazolidone) and compared it with empirical levofloxacin- and bismuth-based quadruple therapy (LBQT) for 14 days as a second-line treatment.

## Methods

### Participants

This study was designed as a multicenter, open-label, randomized, controlled trial and was conducted between May 2016 and June 2019 in the clinics of the First Affiliated Hospital of Nanjing Medical University, Changzhou Second People’s Hospital Affiliated with Nanjing Medical University, and Jinhu County People’s Hospital. Consecutive patients with a failure of clarithromycin-based eradication treatment for *H. pylori* within the previous 6 months were enrolled. Patients were eligible if they had a confirmed *H. pylori* infection based on the ^13^C-urea breath test (UBT). The following exclusion criteria were used: (1) patients who had received *H. pylori* eradication treatment more than once; (2) patients who had been treated with antibiotics, colloidal bismuth pectin, H_2_ receptor inhibitors, or PPIs within the previous 4 weeks; (3) patients with a history of fluoroquinolone drug treatment, particularly levofloxacin; (4) patients with serious diseases, such as severe cardiopulmonary and liver dysfunction; and (5) patients with an allergy to the study drugs. Each patient provided written informed consent, and the study protocol was approved by the ethics committee of each center. The trial was registered at the Chinese Clinical Trial Registry (ChiCTR) with a registration number of ChiCTR1900027743.

### Study groups, trial design, and procedures

According to the consensus on the eradication of *H. pylori* in China [10], we used the following regimens: TBQT consisted of amoxicillin (1000 mg twice daily) + levofloxacin (500 mg once daily) or furazolidone (100 mg twice daily) + esomeprazole (20 mg twice daily) + colloidal bismuth pectin (220 mg twice daily). LBQT consisted of amoxicillin (1000 mg twice daily) + levofloxacin (500 mg once daily) + esomeprazole (20 mg twice daily) + colloidal bismuth pectin (220 mg twice daily). ALEB also consisted of amoxicillin (1000 mg twice daily) + levofloxacin (500 mg once daily) + esomeprazole (20 mg twice daily) + colloidal bismuth pectin (220 mg twice daily). The difference between LBQT and ALEB therapy is that ALEB therapy was based on the results of antibiotic susceptibility testing. LBQT therapy was administered to all patients in the LBQT group, regardless of the results of antibiotic susceptibility testing, and the doctors administering this treatment did not know the results of the susceptibility test. AFEB therapy consisted of amoxicillin (1000 mg twice daily) + furazolidone (100 mg twice daily) + esomeprazole (20 mg twice daily) + colloidal bismuth pectin (220 mg twice daily). All treatment regimens were administered for 14 days.

Before enrollment, patients underwent ^13^C-UBTs for the detection of *H. pylori* infection. Eligible subjects underwent endoscopy for antibiotic susceptibility testing. They were then assigned in a 1:1 ratio to the TBQT group or the LBQT group using a randomized digital table. In the TBQT group, antibiotic selection was based on the results of the susceptibility tests as follows: patients with levofloxacin-sensitive strains were further randomly assigned in a 1:1 ratio to either the ALEB therapy subgroup or the AFEB therapy subgroup. Patients with levofloxacin-resistant strains were assigned to the AFEB therapy subgroup. Successful eradication was evaluated using a ^13^C-UBT performed at least 4 weeks after the treatment ended. Patients in the LBQT group for whom treatment failed in our study were able to undergo their next round of treatment according to the results of the antibiotic susceptibility test performed at the start of this study. Positive results (≥ 4 units) were defined as *H. pylori* treatment failure. Patient compliance and adverse events were assessed through interviews.

### *H. pylori* isolation, culture, and antibiotic susceptibility test

*H. pylori* isolates were obtained from biopsy specimens harvested from the lesser gastric antrum and the greater gastric curvature during endoscopy. Isolation of *H. pylori* and antibiotic susceptibility testing were conducted at the Hangzhou Zhiyuan Medical Inspection Institute. The minimum inhibitory concentrations were defined as follows: amoxicillin ≥ 2 µg/mL, clarithromycin ≥ 1 µg/mL, levofloxacin ≥ 2 µg/mL, furazolidone ≥ 2 µg/mL, and metronidazole ≥ 8 μg/mL [19, 20]. A standard *H. pylori* strain (ATCC43504) was used for quality control.

### CYP2C19 genetic polymorphism

We used the PCR-restriction fragment length polymorphism method to detect the genotypes of variant CYP2C19 alleles (*2, *3, and *17) [21]. DNA was extracted from the gastric mucosal samples by using a QIAamp mini kit (Qiagen, Düsseldorf, Germany). Patients were divided into four groups according to the genotype identified by testing for the CYP2C19 wild-type (CYP2C19 *1) gene and the three mutated alleles (CYP2C19 *2, CYP2C19 *3, and CYP2C19 *17). Patients without a mutation (*1/*1) were defined as the homozygous extensive metabolizer (EM) group, patients with one mutation (*1/*2 or *1/*3) were defined as the heterozygous intermediate metabolizer (IM) group, patients with two mutations (*2/*2, *3/*3, or *2/*3) were defined as the poor metabolizer (PM) group, and patients with the heterozygous CYP2C19 *1/*17 or homozygous CYP2C19 *17/*17 genotype were designated as the ultra-rapid metabolizer (UM) group [22].

### Statistical analysis

The primary outcomes were the eradication rate and side effect rate in the TBQT group and LBQT group. The secondary outcomes were the eradication rate in the ALEB therapy subgroup, combined AFEB therapy subgroup, and LBQT group, and the side effect rate in the ALEB therapy subgroup and combined AFEB therapy subgroup. A P value < 0.05 was considered significant. The eradication rate and patient-reported side effect rate were examined using ITT and PP analyses. Subjects who violated the study protocol, for example, by not taking at least 80% of the treatment drugs, were excluded from the PP analysis. Categorical variables are reported as percentages and were analyzed using the χ^2^ test. Continuous variables were compared between groups using the Student *t* test. A P value < 0.05 was considered significant. The Statistical Package for the Social Sciences software (*version* 25.0; SPSS, Inc., Chicago, IL, USA) version 25.0 was used for the statistical analyses.

## Results

### Participants

**Fig. 1:**
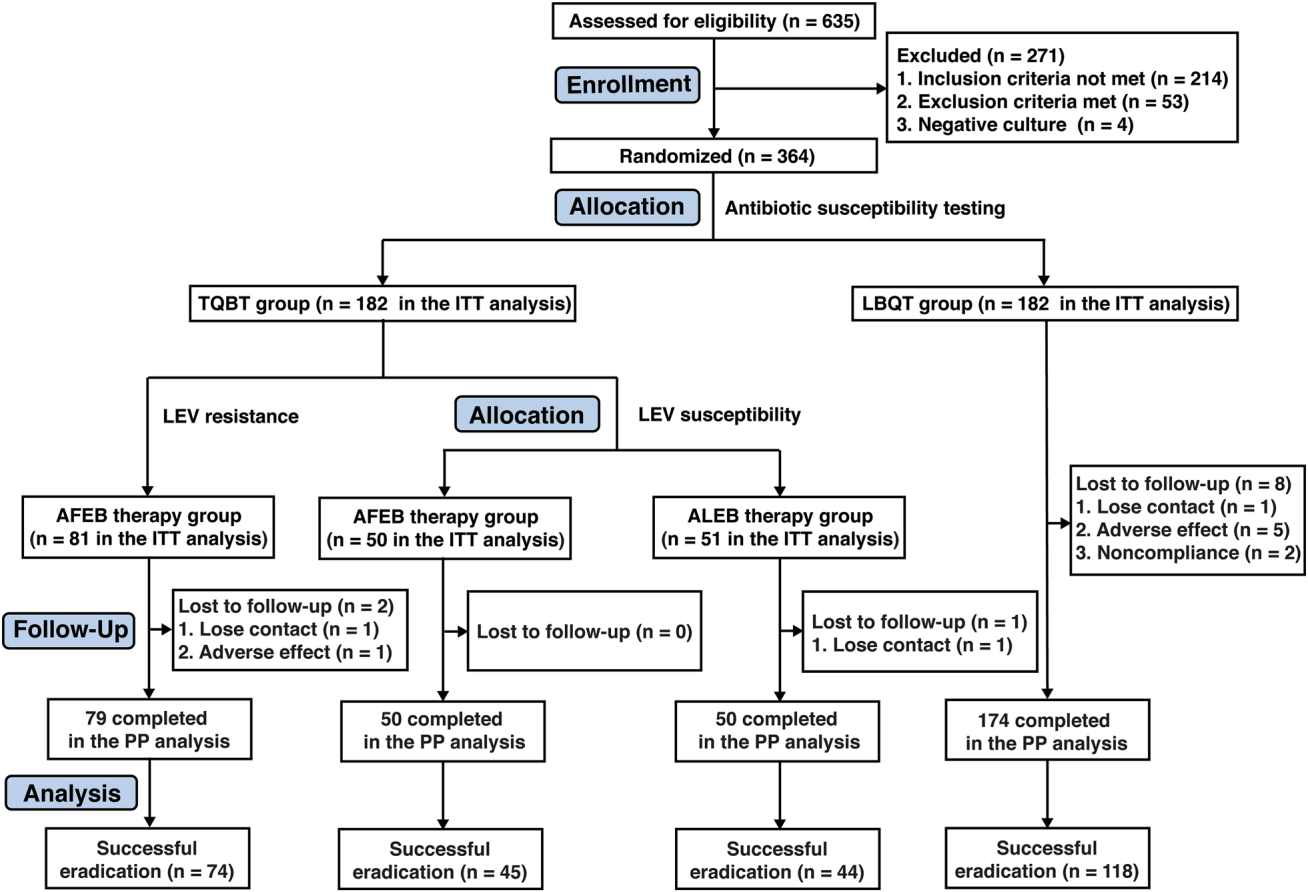
Flow chart of the study design *LEV* levofloxacin, *TBQT* tailored, bismuth-based quadruple therapy, amoxicillin (1000 mg twice daily) + levofloxacin (500 mg once daily) or furazolidone (100 mg twice daily) + esomeprazole (20 mg twice daily) + colloidal bismuth pectin (220 mg twice daily), *LBQT* levofloxacin- and bismuth-based quadruple therapy, amoxicillin (1000 mg twice daily) + levofloxacin (500 mg once daily) + esomeprazole (20 mg twice daily) + colloidal bismuth pectin (220 mg twice daily); ALEB: amoxicillin (1000 mg twice daily) + levofloxacin (500 mg once daily) + esomeprazole (20 mg twice daily) + colloidal bismuth pectin (220 mg twice daily); AFEB: amoxicillin (1000 mg twice daily) + furazolidone (100 mg twice daily) + esomeprazole (20 mg twice daily) + colloidal bismuth pectin (220 mg twice daily), *ITT*, intention-to-treat, *PP* per protocol

Six hundred thirty-five patients were screened during the study period. Of these patients, 271 did not meet the selection criteria, including 93 patients with prior fluoroquinolone use and 106 patients with a history of more than one anti-*H. pylori* treatment regimen; the remaining 364 patients were randomly assigned to the TBQT (n = 182) or LBQT group (n = 182). In the TBQT group, patients without levofloxacin resistance were randomly assigned in a 1:1 ratio to either the ALEB therapy subgroup (n = 51) or the AFEB therapy subgroup (n = 50). Patients in the TBQT group with levofloxacin resistance were assigned to the AFEB therapy group (n = 81). All recruited patients received complete follow-up (Fig. 1).

The baseline characteristics of the participants were well balanced between the groups (Table 1). Eleven of the 364 patients were excluded from the PP analysis; 3 of these patients were lost to follow-up, 6 violated the study protocol because of adverse effects, and 2 patients refused to undergo follow-up ^13^C-UBTs.

**Table 1:** Characteristics of the participants

Characteristic	TBQT-AFEB group (n = 132)	TBQT-ALEB group (n = 50)	LBQT group (n = 182)
Age (yrs), mean ± SD	47.1 ± 11.2	47.2 ± 12.1	48.09 ± 13.6
Male	52% (68/132)	52% (26/50)	47% (86/182)
Smoker	19% (25/132)	20% (10/50)	12% (21/182)
Drinker	20% (26/132)	20% (10/50)	26% (48/182)
BMI (kg/m^2^)	23.21 ± 2.96	22.70 ± 3.20	22.69 ± 3.54
Family history	22% (29/132)	22% (11/50)	19% (34/182)
Peptic ulcer disease	20% (27/132)	20% (10/50)	15% (28/182)
Chronic atrophic gastritis with intestinal metaplasia	36% (47/132)	36% (18/50)	39% (71/182)
Gastrointestinal symptoms	38% (50/132)	38% (19/50)	41% (75/182)
Clarithromycin resistance	57% (75/132)	56% (28/50)	59% (107/182)

*TBQT* tailored, bismuth-based quadruple therapy, amoxicillin + levofloxacin or furazolidone + esomeprazole + colloidal bismuth pectin, *LBQT* levofloxacin- and bismuth-based quadruple therapy, amoxicillin + levofloxacin + esomeprazole + colloidal bismuth pectin, *BMI* body mass index

### Antibiotic resistance

**Fig. 2:**
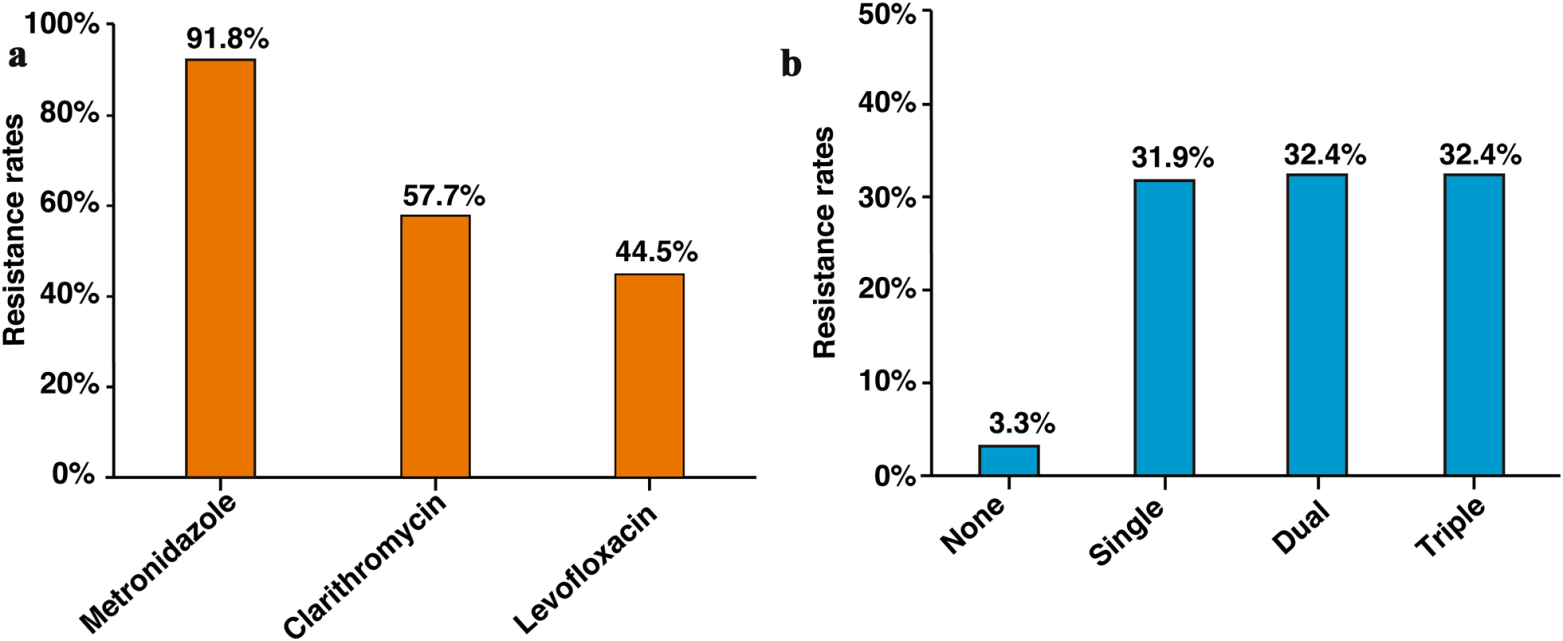
Antibiotic resistance rates **a** The metronidazole-, clarithromycin-, and levofloxacin-resistance rates in the total population. **b** Multidrug-resistant infections. None, Single, Dual, and Triple indicate no, single, dual, and triple resistance to clarithromycin, levofloxacin, and metronidazole in the TBQT group, respectively. Multidrug-resistant strains were predominant, with 64.8% of strains showing dual or triple resistance

The metronidazole-, clarithromycin-, and levofloxacin-resistance rates were 91.8% (334/364), 57.7% (210/364), and 44.5% (162/364), respectively (Fig. 2a). In our population, multidrug-resistant strains were predominant. Only 3.3% (12/364) of *H. pylori* isolates were susceptible to all three tested antibiotics; the remaining 96.7% of *H. pylori* isolates showed single, dual, or triple resistance to clarithromycin, levofloxacin, and metronidazole. Single antibiotic resistance was most commonly observed for metronidazole (91.8%), while dual antibiotic resistance (32.4%) was most commonly observed for metronidazole and clarithromycin. The pooled prevalence of clarithromycin, metronidazole, and levofloxacin resistance was 32.4% (118/364; Fig. 2b). In addition, the total clarithromycin and levofloxacin resistance rate among strains with dual (clarithromycin and levofloxacin, n = 12/118) or triple (clarithromycin, levofloxacin and metronidazole, n = 118/118) resistance was 35.7% (130/364).

### Clinical efficacy

The eradication rates in the TBQT and LBQT groups were 89.6% and 64.8% (P < 0.001) in the ITT analysis and 91.1% and 67.8% (P < 0.001) in the PP analysis, respectively (Table 2). The eradication rate in the TBQT group was significantly higher than in the LBQT group (P < 0.001).

**Table 2:** *H. pylori* eradication rates in the TBQT and LBQT groups

Eradication rate	TBQT group (n = 182)	LBQT group (n = 182)	P value
ITT analysis	163/182 (89.6%)	118/182 (64.8%)	< 0.001
PP analysis	163/179 (91.1%)	118/174 (67.8%)	< 0.001

*TBQT* tailored, bismuth-based quadruple therapy, amoxicillin + levofloxacin or furazolidone + esomeprazole + colloidal bismuth pectin, *LBQT* levofloxacin- and bismuth-based quadruple therapy. amoxicillin + levofloxacin + esomeprazole + colloidal bismuth pectin, *ITT* intention‐to‐treat, *PP* per protocol

### Clinical efficacy in the TBQT group

**Fig. 3:**
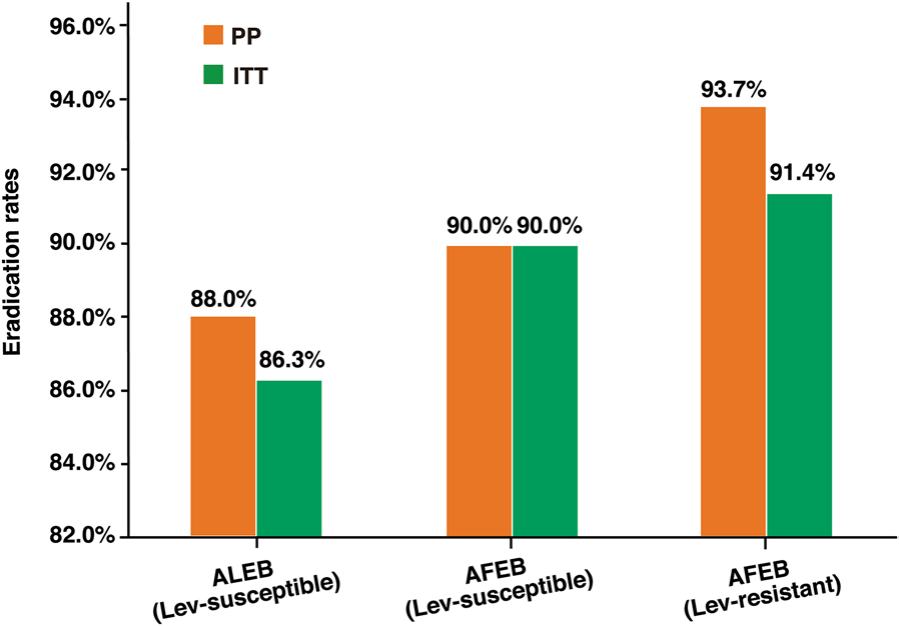
*H. pylori* eradication rates in the TBQT group ALEB/AFEB: amoxicillin (1000 mg twice daily) + levofloxacin (500 mg once daily) or furazolidone (100 mg twice daily) + esomeprazole (20 mg twice daily) + colloidal bismuth pectin (220 mg twice daily) Lev: levofloxacin Clinical efficacies in the levofloxacin-susceptible group (ALEB therapy subgroup and AFEB therapy subgroup) and levofloxacin-resistant group (AFEB therapy subgroup) according to the ITT and PP analyses

The eradication rates in the levofloxacin-susceptible ALEB therapy subgroup and AFEB therapy subgroup were 88.0% and 90.0% (P = 0.75), respectively, in the PP analysis and 86.3% and 90.0% (P = 0.56), respectively, in the ITT analysis (Fig. 3). There was no significant difference in the eradication rate among the three subgroups in the TBQT group in the ITT (P = 0.64) and PP analyses (P = 0.52). In all, in the levofloxacin-susceptible group, the ALEB and AFEB regimens achieved similar efficacy rates.

### Clinical efficacy in the ALEB therapy subgroup, AFEB therapy subgroup, and LBQT group

The eradication rates in the ALEB therapy subgroup and LBQT group were 86.3% and 64.8% (P = 0.003), respectively, in the ITT analysis and 88.0% and 67.8% (P = 0.005), respectively, in the PP analysis (Table 3). The eradication rates in the combined AFEB therapy subgroup (i.e., including all patients in the levofloxacin-susceptible and levofloxacin-resistant groups who received AFEB therapy) and LBQT group were 90.8% and 64.8% (P = 0.000), respectively, in the ITT analysis and 92.2% and 67.8% (P = 0.000), respectively, in the PP analysis. A significant difference in the eradication rate was not observed between the ALEB therapy subgroup and the combined AFEB therapy subgroup in the ITT (P = 0.366) and PP analyses (P = 0.371).

**Table 3:** *Helicobacter pylori* eradication rates in the ALEB therapy subgroup, combined AFEB therapy subgroup, and LBQT group

Eradication rate	ALEB therapy subgroup (n = 51)	Combined AFEB therapy subgroup (n = 131)	LBQT group (n = 182)
ITT analysis	44/51 (86.3%)	119/131 (90.8%)	118/182 (64.8%)
PP analysis	44/50 (88.0%)	119/129 (92.2%)	118/174 (67.8%)

*ALEB (susceptibility-guided therapy)* amoxicillin + levofloxacin + esomeprazole + colloidal bismuth pectin, *LBQT* levofloxacin‐ and bismuth-based quadruple therapy not guided by antibiotic susceptibility, amoxicillin + levofloxacin + esomeprazole + colloidal bismuth pectin, *AFEB* amoxicillin + furazolidone + esomeprazole + colloidal bismuth pectin, *ITT* intention‐to‐treat, *PP* per protocol

### Adverse events

The safety analysis included 361 participants. Three patients were lost to follow-up. The incidence of adverse events in the TBQT and LBQT groups was 40.0% (72/180) and 45.9% (83/181; P = 0.26), respectively. One participant in the levofloxacin-resistant group who received AFEB therapy and five participants in the LBQT group violated the study protocol due to abdominal discomfort, nausea, and dizziness. The incidence of adverse events did not significantly differ between the groups (Additional file 1: Table S1). In the TBQT group, 132 patients received AFEB therapy (i.e., including all patients in the levofloxacin-susceptible and levofloxacin-resistant groups who received AFEB therapy), and the side effects of AFEB therapy were not more severe than those of LBQT and ALEB therapy (Additional file 1: Table S2).

### Analysis stratified according to CYP2C19 polymorphisms

Of the 364 samples, 28 were insufficient. Thus, 336 samples were subjected to the analysis of CYP2C19 polymorphisms, and 0.9% (3/336) of patients were UMs, 46.4% (156/336) were EMs, 39.3% (132/336) were IMs, and 13.4% (45/336) were PMs (Supplementary Figure S1).

The eradication rates among EMs, IMs, and PMs were 78.8% (123/156), 81.1% (107/132), and 84.4% (38/45), respectively. As shown in Additional file 1: Table S3, no significant difference in the eradication rate was observed among the different CYP2C19 polymorphism-based subgroups (P = 0.69).

## Discussion

Physicians must consider the patient’s previous exposure to antibiotics to optimize the management of *H. pylori* treatment. In this prospective, randomized trial, clarithromycin-containing therapy had been administered to the included patients as a first-line treatment. We therefore selected AFEB and ALEB therapy as second-line regimens for *H. pylori*-positive patients. We confirmed that TBQT (AFEB or ALEB) was more effective than LBQT under conditions of a high prevalence of antibiotic resistance, according to both the ITT (86.3% vs. 64.8%) and PP analyses (88.0% vs. 67.8%). However, one systematic review has suggested that the evidence was too limited to support the generalized use of susceptibility-guided therapy as a rescue treatment for *H. pylori* infection because of unclear antibiotic resistance [18]. In our study, the levofloxacin-resistance rate was 44.5%, consistent with previously reported rates of 34.5–54.8% in China [23]. In addition, the prevalence of dual clarithromycin and levofloxacin resistance in the whole cohort was 35.7%. Thus, if levofloxacin- or clarithromycin-containing quadruple therapy was to be administered as a second-line therapy to patients who had previously experienced failure of clarithromycin-containing treatment, the possibility of treatment failure would be very high. However, the use of an antibiotic susceptibility-guided approach helped achieve more satisfactory results with second-line levofloxacin-containing treatment, with eradication rates exceeding 85% in our study. In patients without levofloxacin resistance, the ALEB regimen achieved an efficacy similar to AFEB therapy. In general, most guidelines strongly recommend susceptibility-guided therapy under the circumstances of prior inappropriate antibiotic use and widespread resistance development [15]. The main factor resulting in failure of *H. pylori* eradication treatment is presumed to be antibiotic resistance [24].

Cure rates greater than 90–95% should be expected with antimicrobial therapy for *H. pylori* infection [2]. Compared to other antibiotics used as second-line treatments, metronidazole has achieved excellent cure rates (> 90%) in Japan [18, 25]. The success rate of tetracyclines or clarithromycin is also greater than 90% in Taiwan and other regions [26–29]. However, second-line *H. pylori* treatments that achieve cure rates greater than 90% are extremely heterogeneous [18]. The administration of metronidazole and clarithromycin-containing therapy as a rescue regimen was expected to be inefficient in our study because of the high antibiotic resistance rate. In our study population, when using the susceptibility-guided approach, only AFEB therapy yielded efficacy rates greater than 90% in patients with a history of clarithromycin treatment. Levofloxacin, a fluoroquinolone-based antibiotic, has a lower resistance rate than clarithromycin [5]. Bismuth- and levofloxacin-containing quadruple therapy should be reserved as an effective (≥ 90% success rate) second-line strategy for patients who have experienced one treatment failure [30, 31]. However, levofloxacin resistance has increased since the restriction of macrolides [32], and thus it is markedly less effective in fluoroquinolone-containing triple therapies than other agents [33]. In our previous study, we compared the efficacy of levofloxacin-based regimens as first-line anti-*H. pylori* treatments and found a low success rate (78%) [34]. Hence, AFEB therapy might be the preferred regimen in regions where bacterial susceptibility data are not available to avoid increased levofloxacin resistance [35, 36]. Furazolidone is an effective drug for *H. pylori* eradication; it has a low resistance rate against *H. pylori* and is available in many regions [7]. According to some studies, furazolidone-based quadruple therapy, which has an 88.2% eradication rate, is an efficacious rescue strategy in patients with a previous eradication therapy failure [35].

In addition, we used esomeprazole in this study, and previous study have reported that CYP2P19 polymorphisms do not influence *H. pylori* eradication rates when esomeprazole or rabeprazole is administered [37]. Consistent with the previous study, we did not observe any significant effect of CYP2C19 polymorphisms on the efficacy of eradication treatment (Additional file 1: Figure S1 and Table S3).

This study has some limitations. First, the relatively small sample size (n = 51) in the ALEB therapy group prevented us from clearly explaining the efficacy of levofloxacin. Further studies will be needed to reach an evidence-based conclusion. Second, the patients from the TBQT group were assigned to subgroups according to the results of the antibiotic susceptibility tests; thus, this study was not a completely double-blind study. Fourth, the low BMI (< 24 kg/m^2^) of our study population may produce a possible selection bias; the next step will be to perform further screening of a patient population with a high BMI.

## Conclusions

In conclusion, second-line levofloxacin-containing quadruple therapy exhibited unacceptable therapeutic efficacy in patients with a failure of clarithromycin-based first-line treatment. The administration of a second-line levofloxacin-containing treatment using a susceptibility-guided approach, however, displayed acceptable efficacy. AFEB therapy was recommended as a rescue regimen in regions where bacterial susceptibility data were not available to avoid increased levofloxacin resistance.

## Supplementary information

**Additional file 1: Figure S1.** CYP2C19 polymorphism rates. **Table S1.** Adverse events in patients undergoing eradication therapy. **Table S2.** Adverse events in patients undergoing eradication therapy. **Table S3.** Analysis according to CYP2C19 polymorphisms.

## Data Availability

The datasets used and/or analyzed during the current study are available from the corresponding author upon reasonable request.

## Abbreviations

*H. pylori*: Helicobacter pylori
PPIs: Proton pump inhibitors
ITT: Intention-to-treat
PP: Per protocol
CBQT: Clarithromycin- and bismuth-based quadruple therapy
TBQT: Tailored bismuth-based quadruple therapy
LBQT: Levofloxacin- and bismuth-based quadruple therapy, amoxicillin (1000 mg twice daily) + levofloxacin (500 mg once daily) + esomeprazole (20 mg twice daily) + colloidal bismuth pectin (220 mg twice daily)
ALEB: Amoxicillin (1000 mg twice daily) + levofloxacin (500 mg once daily) + esomeprazole (20 mg twice daily) + colloidal bismuth pectin (220 mg twice daily)
AFEB: Amoxicillin (1000 mg twice daily) + furazolidone (100 mg twice daily) + esomeprazole (20 mg twice daily) + colloidal bismuth pectin (220 mg twice daily)
UBT: Urea breath test
EM: Extensive metabolizer
IM: Intermediate metabolizer
PM: Poor metabolizer
UM: Ultra-rapid metabolizer
