# Supplementary material for: Efficacy of tailored second-line therapy of Helicobacter pylori eradication in patients with clarithromycin-based treatment failure: a multicenter prospective study
Source: Gut Pathog. 2020 Aug 29;12:39. doi: 10.1186/s13099-020-00378-1 (PMC7456506; doi:10.1186/s13099-020-00378-1)
Supplement: Supplementary file 1 — Additional file 1: Figure S1. CYP2C19 polymorphism rates. Table S1. Adverse events in patients undergoing eradication therapy. Table S2. Adverse events in patients undergoing eradication therapy. Table S3. Analysis according to CYP2C19 polymorphisms. [file 13099_2020_378_MOESM1_ESM.docx]

**Additional file 1**

**Figure S1. CYP2C19 polymorphism rates**


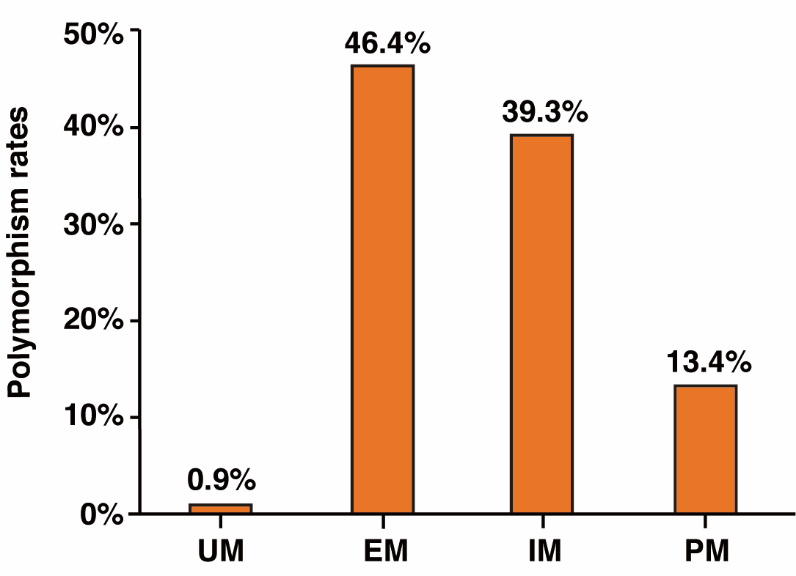


UM: ultra-rapid metabolizer; EM: extensive metabolizer; IM: intermediate metabolizer; PM: poor metabolizer

**Table S1. Adverse events in patients undergoing eradication therapy**

| Adverse events | TBQT group  (*n* = 180) | | LBQT group  (*n* = 181) | *P* value |
| --- | --- | --- | --- | --- |
| Abdominal discomfort | | 11 (6.1%) | 15 (8.3%) |  |
| Nausea/vomiting | | 14 (7.8%) | 17 (9.4%) |  |
| Diarrhea | | 44 (24.4%) | 21 (11.6%) |  |
| Dyspepsia | | 13 (7.2%) | 32 (17.7%) |  |
| Taste disturbance | | 34 (18.9%) | 27 (14.9%) |  |
| Dry mouth | | 2 (1.1%) | 6 (3.3%) |  |
| Constipation | | 4 (2.2%) | 7 (3.9%) |  |
| Itching | | 3 (1.7%) | 1 (0.6%) |  |
| Dizziness | | 2 (1.1%) | 6 (3.3%) |  |
| Any adverse event | | 72 (40.0%) | 83 (45.9%) | 0.26 |

TBQT: tailored, bismuth-based quadruple therapy; LBQT: levofloxacin- and bismuth-based quadruple therapy

**Table S2. Adverse events in patients undergoing eradication therapy**

| Adverse events | Combined AFEB therapy (*n* = 130) | | LBQT and ALEB group  (*n* = 231) | *P* value |
| --- | --- | --- | --- | --- |
| Abdominal discomfort | | 8 (6.2%) | 18 (7.8%) |  |
| Nausea/vomiting | | 12 (9.2%) | 19 (8.2%) |  |
| Diarrhea | | 40 (30.8%) | 25 (10.8%) |  |
| Dyspepsia | | 9 (6.9%) | 36 (15.6%) |  |
| Taste disturbance | | 29 (22.3%) | 32 (13.9%) |  |
| Dry mouth | | 3 (2.3%) | 5 (2.2%) |  |
| Constipation | | 4 (3.1%) | 7 (3.0%) |  |
| Itching | | 2 (1.5%) | 2 (0.8%) |  |
| Dizziness | | 2 (1.5%) | 6 (2.6%) |  |
| Any adverse event | | 58 (44.6%) | 96 (41.6%) | 0.57 |

ALEB/AFEB: amoxicillin + levofloxacin or furazolidone + esomeprazole + colloidal bismuth pectin ; LBQT: levofloxacin- and bismuth-based quadruple therapy

**Table S3. Analysis according to CYP2C19 polymorphisms**

| CYP2C19 polymorphism | Success | Failure | Eradication rate |
| --- | --- | --- | --- |
| EMs | 123 | 33 | 78.8% |
| IMs | 107 | 25 | 81.1% |
| PMs | 38 | 7 | 84.4% |
| *P* value |  |  | 0.69 |

EM: extensive metabolizer; IM: intermediate metabolizer; PM: poor metabolizer
